# Potential Policy Targets to Reduce Vaping Among Youths

**DOI:** 10.1001/jamanetworkopen.2024.51685

**Published:** 2024-12-18

**Authors:** Andrew F. Brouwer, Jamie Hartmann-Boyce, Abigail S. Friedman

**Affiliations:** 1 Department of Epidemiology, University of Michigan, Ann Arbor; 2 Department of Health Promotion and Policy, University of Massachusetts, Amherst; 3 Department of Health Policy and Management, Yale School of Public Health, New Haven, Connecticut

## Abstract

This cross-sectional study uses Population Assessment of Tobacco and Health study data to estimate the association between different policy targets and electronic nicotine delivery system use among US youths compared with adults.

## Introduction

Over 25% of US residents live in regions with restrictions on sales of flavored electronic nicotine delivery systems (ENDS), which are typically enacted to reduce youth vaping. Concurrently, randomized clinical trials (RCTs) suggest that adults who smoke are more likely to quit combustible cigarettes with the aid of ENDS than without, although effectiveness outside research contexts remains contested.^1,2^ If RCT evidence generalizes, regulations restricting ENDS characteristics to protect youths might reduce smoking cessation among the 14.5% of adults smoking combustible tobacco.^3^ Clarifying differential preferences for ENDS characteristics—particularly flavors and device types—by age may help policy makers identify policy targets that align with their goals, whether targeting the maximum number of vaping youths, minimizing the ratio of adults to youths targeted, or meeting another objective.

## Methods

Using cross-sectional data from wave 7 (January 2022 to April 2023) of the nationally representative Population Assessment of Tobacco and Health (PATH) study,^4^ we estimated weighted prevalences (with 95% CIs) of past 30-day ENDS use among youths (12-17 years), young adults (18-24 years), and adults (25-54 years). Older adults were excluded because current data may not be representative for that cohort under counterfactual ENDS policies (as tobacco product use most affects mortality at ages ≥55 years). The University of Michigan Institutional Review Board approved this secondary analysis. PATH participants provided informed consent. This cross-sectional study followed the STROBE reporting guideline.

Calculations were repeated for participants who had ever smoked at least 100 cigarettes in their lifetime. For each subgroup, we calculated the fraction of those reporting past 30-day vaping who used disposable, flavored (other than tobacco), and flavored disposable ENDS. To estimate the precision with which a hypothetical policy banning flavored or disposable ENDS would constrain adult vs youth product choice, we calculated the ratio of the number of ever-smoking adults using ENDS with a given characteristic to the number of youths using that ENDS type. Analyses were performed with R, version 4.4 (R Project for Statistical Computing).

## Results

There were 10 650 youth, 10 310 young adult, and 13 344 adult participants (unweighted counts). Almost all participants who vaped in the past 30 days reported using flavored ENDS (98.3% of youths, 96.9% of young adults, and 90.2% of adults who vaped) (Table). The pattern differed for disposable ENDS use: 73.6% of youths and 76.4% of young adults who vaped used disposable products vs 61.4% of adults. Almost all disposable ENDS use was flavored. Patterns of flavored and disposable use were largely similar for those who reported having smoked at least 100 cigarettes. These estimates suggest that flavor prohibitions would constrain product choice for 5.6 ever-smoking adults for each affected vaping youth (7 540 254/1 339 654), compared with 5.0 for a ban on disposable ENDS (5 011 937/1 002 826). A ban on disposables is estimated to affect 11.2% (2 528 317) fewer ever-smoking adults per 1 vaping youth than a flavor ban; however, it would also target 25.1% (336 828) fewer vaping youths. Combining young adults with adults amplified these trade-offs.

**Table.** Prevalence of Past 30-Day Vaping and Product Characteristics by Age Group^a^

Vaping in past 30 d	All participants			Ever (current and former) cigarette use by age^b^		
	12-17 y (n = 26 248 359)	18-24 y (n = 29 419 729)	25-54 y (n = 126 124 932)	12-17 y (n = 103 981)	18-24 y (n = 2 981 234)	25-54 y (n = 47 934 766)
Vaped ENDS	1 362 493 (5.2 [4.8-5.6])	5 217 150 (17.7 [16.9-18.6])	10 154 088 (8.1 [7.2-8.9])	73 238 (70.4 [55.5-85.4])	1 704 539 (57.2 [53.2-61.2])	8 484 552 (17.7 [16.3-19.1])
Among those who vaped ENDS						
Used flavored ENDS	1 339 654 (98.3 [97.5-99.2])	5 054 884 (96.9 [95.9-97.9])	9 159 457 (90.2 [88.1-92.3])	70 351 (96.1 [88.3-103.8])	1 602 283 (94.0 [90.8-97.2])	7 540 254 (88.9 [86.5-91.2])
Used disposable ENDS	1 002 826 (73.6 [69.7-77.5])	3 987 415 (76.4 [73.9-79.0])	6 236 808 (61.4 [58.4-64.5])	60 113 (82.1 [67.5-96.7])	1 168 355 (68.5 [62.9-74.2])	5 011 937 (59.1 [55.3-62.9])
Used flavored disposable ENDS	993 602 (72.9 [68.8-77.0])	3 885 437 (74.5 [71.5-77.5])	5 881 673 (57.9 [54.5-61.3])	58 332 (79.6 [62.8-96.5])	1 096 330 (64.3 [58.4-70.2])	4 672 880 (55.1 [50.9-59.2])

Abbreviation: ENDS, electronic nicotine delivery systems.

^a^ Values are presented as No. (% [(95% CI]) of respondents. Sample-weighted mean percentages and 95% CIs are calculated by age group from the Population Assessment of Tobacco and Health study wave 7 data (fielded from January 2022 to April 2023). Counts are sample weighted to reflect the proportion of US residents represented by each subgroup.

^b^ Reported having smoked at least 100 cigarettes in their lifetime.

## Discussion

In this cross-sectional study, we estimated the number of US individuals who would lose access to their preferred ENDS product under different policy targets. The findings suggest that flavor bans would have consequences for not only nearly all youths who vape but also most adults who have used or may use ENDS to quit cigarettes. In contrast, banning disposable ENDS is estimated to affect 11.2% (2 528 317) fewer ever-smoking adults per vaping youth than a flavor ban, but would constrain 25.1% (336 828) fewer youths who vape.

This study has several limitations. First, because most US restrictions on flavored ENDS apply to both menthol and nonmenthol flavors, we did not differentiate these flavor types. Future work should explore the potential of more specific flavor targeting (eg, menthol vs desserts). Similarly, distinguishing use patterns by other product characteristics (eg, nicotine formulation) may be valuable. Finally, banning different product characteristics could elicit different responses (eg, cessation vs product switching) and outside benefits (eg, disposable ENDS account for over half of the US ENDS market and bring substantial environmental burdens^5,6^). Further research is needed to project outcomes of these policies and to help policy makers navigate the trade-offs associated with different policy targets.

## Notes

Supplement.
Data Sharing Statement
