# Supplementary material for: Potential Policy Targets to Reduce Vaping Among Youths
Source: JAMA Netw Open. 2024 Dec 18;7(12):e2451685. doi: 10.1001/jamanetworkopen.2024.51685 (PMC11656260; doi:10.1001/jamanetworkopen.2024.51685)
Supplement: Supplement. — Data Sharing Statement [file jamanetwopen-e2451685-s001.pdf]

Data Sharing Statement

Brouwer. Potential Policy Targets to Reduce Vaping Among Youths. *JAMA Netw Open*. Published December 18, 2024. doi:10.1001/jamanetworkopen.2024.51685

Data
Data available: No

Additional Information
Explanation for why data not available: This study makes use of restricted data from the Population Assessment of Tobacco and Health (https://doi-org.proxy.lib.umich.edu/10.3886/ICPSR36231.v40). Instructions for accessing restricted use data through the National Addition & HIV Data Archive Program (NAHDAP) are available at the listed website.
